# m^6^A mRNA Methylation in Hematopoiesis: The Importance of Writing, Erasing, and Reading

**DOI:** 10.3390/cells14171388

**Published:** 2025-09-05

**Authors:** Antonia-Gerasimina Vasilopoulou, Eleni Kalafati, Ekati Drakopoulou, Nicholas P. Anagnou

**Affiliations:** Laboratory of Cell and Gene Therapy, Centre of Basic Research, Biomedical Research Foundation of the Academy of Athens (BRFAA), 11527 Athens, Greece; antoniage.vasilopoulou@gmail.com (A.-G.V.); edrakopoulou@bioacademy.gr (E.D.)

**Keywords:** epitranscriptomics, m^6^A modification, m^6^A regulators, hematopoietic stem cells, acute myeloid leukemia (AML), erythropoiesis, ineffective erythropoiesis, thalassemia, hemoglobin H disease

## Abstract

Over recent years, epitranscriptomic research has provided a new layer of gene regulation during hematopoietic development and aberrant hematopoiesis. Among the 170 identified RNA chemical marks, N^6^-methyladenosine (m^6^A) is the most abundant in eukaryotic cells and plays a critical role in various biological processes. This dynamic modification is regulated by a series of methyltransferases, demethylases, and m^6^A binding proteins, known as writers, erasers, and readers, respectively. Emerging evidence suggests that m^6^A modification and its regulators are involved in every aspect of normal hematopoietic development, from the emergence of hematopoietic stem cells to the generation of mature blood cells. Also, it has been established that abnormal expression of m^6^A regulators is implicated in the initiation of blood diseases. In this review, we summarize the latest findings regarding the role of m^6^A in erythropoiesis and highlight its implications in the pathophysiology of hemoglobin disorders.

## 1. Introduction

### The Biochemistry of N^6^-Methyladenosine RNA Modification

During the last few decades, advances in epigenetic research have provided novel insights into the mechanisms that control either normal hematopoiesis or lead to blood disorders. The production and development of hematopoietic stem cells (HSCs) is a complex process regulated at multiple levels by epigenetic and epitranscriptomic factors, including DNA methylation, histone modification, chromatin modification, and RNA methylation [1,2,3]. So far, a significant number of post-transcriptional modifications of mRNA have been identified, among which the nitrogen-6-methyladenosine (m^6^A) is the most common. The m^6^A marking is a dynamic RNA modification, abundant in endogenous eukaryotic messenger RNAs [4]. Although m^6^A was first discovered in the early 1970s [5,6], only recent advances in sequencing technologies have allowed for mapping the m^6^A-modified sites in mammalian cells and tissues and thus have begun deciphering its role in physiological processes and disease [7,8]. Marks of m^6^A occurring in adenines of RRACH sequence (R=G or A; H=A, C, or U) are in abundance in the 3′ untranslated regions (3′ UTRs), in long internal exons, and around stop codons [7]. The location of these m^6^A marks is functionally decisive. For instance, when present within the 3′ UTRs or near stop codons, transcript turnover is usually promoted [4,9], while enrichment of m^6^A in the 5′ UTR region facilitates cap-independent translation initiation by recruiting eukaryotic initiation factor 3 (eIF3), a mechanism particularly important under conditions of cellular stress [10].

**Figure 1:**
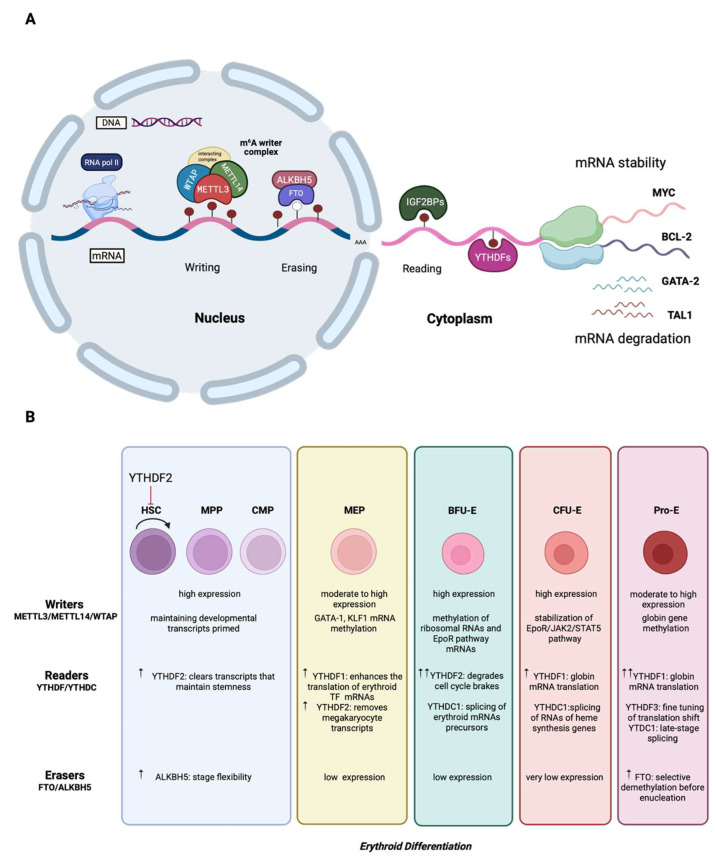
Features of the m^6^A RNA modification mechanism and its role on hematopoietic stem cell (HSC) development. (**A**) m^6^A methylation occurs in the nucleus by the MTase complex (writers), consisting of METTL3, which is the catalytic subunit; METTL14; WTAP; and other cofactors, and is erased by the demethylases ALKBH5 and FTO (erasers). The fate of the m^6^A-tagged mRNAs is determined by the m^6^A-binding proteins known as readers (e.g., YTH family of proteins; IGF2BP1, 2, and 3; HNRNPA2B1), whose actions can confer either mRNA stability or induce mRNA degradation. Created in BioRender. Vasilopoulou, A-G. (2025) https://BioRender.com/u0vton0 (accessed on 26 August 2025). (**B**) Recent findings suggest that m^6^A regulators are key players during HSC development and erythroid differentiation. The expression pattern and the role of several writers (METTL3/METTL14/WTAP), readers (YTHDF and YTHDC), and erasers (FTO and ALKBH5) during the different stages of human erythropoiesis are shown in detail. Circular arrow denotes self-renewal and proliferation at the specific stage. Upward arrows indicate increased expression. HSC, hematopoietic stem cell; MPP, multipotent progenitor; CMP, common myeloid progenitor; MEP, megakaryocyte-erythroid progenitor; GATA-1, GATA binding protein 1; KLF1, Krüppel-like factor 1; BFU-E, burst-forming unit-erythroid; CFU-E, colony-forming unit-erythroid; JAK2, Janus kinase 2; STAT5, signal transducer and activator of transcription 5; Pro-E, proerythroblast. Created in BioRender. Vasilopouou, A-G. (2025) https://BioRender.com/3l0ezm9 (accessed on 26 August 2025).

The m^6^A marks are deposited in the nucleus by the m^6^A methyltransferase (MTase) complex (writers), consisting of methyltransferase-like 3 (METTL3), which is the catalytic subunit; methyltransferase-like 14 (METTL14), which provides structural support and RNA-binding stabilization; Wilm’s tumor 1-associating protein (WTAP), which acts as a regulatory subunit [11]; and other cofactors. Although WTAP lacks inherent methylation activity, it interacts with the METTL3-METTL14 heterodimer to substantially affect the efficiency and specificity of m^6^A deposition [12]. Alongside the core components of the MTase complex, several co-factors modulate the efficiency and selectivity of m^6^A deposition. Specifically, vir-like m^6^A methyltransferase (VIRMA, also known as KIAA1429) guides the writer complex to methylation sites, enriched in 3′ UTR and stop codons [13]; RNA-binding motif protein 15 (RBM15) is a major RNA-binding adaptor that recruits the MTase complex to specific targets [14]; E3 ubiquitin-protein ligase Hakai (CBLL1) contributes to the assembly and stability of the writer complex [13]; and zinc finger CCCH domain-containing protein 13 (ZC3H13) stabilizes the interaction among WTAP, VIRMA, and RBM15 [15]. While the majority of m^6^A modifications are deposited by the MTase complex, methyltransferase-like 16 (METTL16) has also emerged as a versatile regulator of RNA processing [16,17]. In the nucleus, METTL16 deposits m^6^A into mRNA targets, whereas in the cytoplasm it promotes mRNA translation through an m^6^A-independent mechanism [17]. This RNA methylation process is reversed by the m^6^A demethylases (erasers), fat mass and obesity-associated protein (FTO) [18] and α-ketoglutarate-dependent dioxygenase ALKB homolog 5 (ALKBH5) [19], which thereby reduce the global m^6^A levels [16,17]. Finally, m^6^A-binding proteins, known as readers, are located both in the nucleus and in the cytoplasm, and determine the fate of the m^6^A-modified transcripts by affecting mRNA nuclear export, splicing, stability, and translation efficiency, as depicted in Figure 1. The best-described readers belong to the YT521-B homology (YTH) domain family, which includes the YTHDF1, YTHDF2, YTHDF3, YTHDC1, and YTHDC2 proteins [20]. Among them, the YTHDF proteins located in the cytoplasm are primarily associated with the regulation of mRNA stability and translation, with YTHDF2 accelerating transcript degradation [9] and YTHDF1 and YTHDF3 promoting translation and cooperating in maintaining mRNA turnover balance [21]. In the nucleus, YTHDC1 has been shown to affect alternative splicing of the m^6^A-modified mRNAs by recruiting splicing factors [22]. Most recently, insulin-like growth factor 2 mRNA-binding proteins (IGF2BPs) and proteins of the heterogeneous nuclear ribonucleoprotein (HNRNP) family were found to impact the stability and translation of targeted mRNAs, as well as the alternative splicing and microRNA processing, respectively [20].

Regarding the biological role of m^6^A, accumulating evidence suggests that it is involved in a broad spectrum of biological processes, such as tissue development, circadian rhythm, apoptosis, autophagy, and tumorigenesis [23,24,25,26,27]. For instance, dynamic changes in the topology of the m^6^A marks between fetal and adult stages have been reported, with these modifications being closely linked to genes governing embryonic development and tissue-specific functions [23]. Furthermore, it has been reported that m^6^A modification regulates circadian rhythms by modulating the processing and stability of key clock gene transcripts. Inhibition of the m^6^A methyltransferase METTL3 disrupts RNA processing and lengthens the circadian period [24]. In addition, m^6^A has been shown to influence cell fate decisions by modulating apoptotic pathways, for example, through the regulation of transcripts encoding pro- and anti-apoptotic proteins, thereby affecting cell survival under stress conditions [26]. It also contributes to autophagy by controlling the methylation status and translation efficiency of autophagy-related genes [25]. Regarding its implication in tumorigenesis, several studies suggest that aberrant expression of m^6^A regulators disrupts normal gene expression programs. These changes can stabilize oncogenic transcripts; destabilize tumor suppressors; and ultimately drive malignant transformation, progression, and therapeutic resistance [27].

Recent studies revealed that m^6^A-methyladenosine RNA modification plays a crucial role in hematopoietic cell fate decisions by regulating both self-renewal and HSC differentiation [28,29,30,31]. This review highlights insights into how m^6^A RNA modification regulates hematopoiesis, emphasizing its critical role in erythropoiesis and its impact on the development of hemoglobin disorders.

## 2. The Role of m^6^A RNA Methylation in Normal Hematopoiesis and Disease

### 2.1. HSCs and Endothelial-to-Hematopoietic Transition

The blood system is comprised of more than ten different blood cell types, termed lineages, with numerous functions, from immune surveillance and defense to O_2_ transport and wound healing [32]. All blood cell types are generated from HSCs that reside mostly in the bone marrow, a major site of adult hematopoiesis [32]. The primary stages of embryonic development of blood tissue literally take place in the absence of any blood cells [33]. The sites of the de novo hematopoietic stem and progenitor cell (HSPC) generation, as well as the maintenance and expansion, are constantly changing in the developing embryo [34]. The first hematopoietic cells are generated in the extraembryonic yolk sac, before the first heartbeat, and later in the allantois and placenta [34]. All adult and many of the later embryonic blood cells are generated from HSCs. However, there is a transient population of primitive blood cells in the early embryo generated from the endothelium through a highly conserved process known as endothelial-to-hematopoietic transition (EHT) [35,36]. Molecular signaling by Notch ligands, hedgehog, bone morphogenetic protein (BMP), vascular endothelial growth factor (VEGF), and nitric oxide signaling play a crucial role in the induction of the hemogenic program in endothelial cells [34].

During EHT, cells undergo significant morphological changes, including the breaking of tight connections with surrounding endothelial cells and rounding up, before escaping into the bloodstream [37]. A complex interplay of critical transcription factors, such as runt-related transcription factor-1 (RUNX1) and c-MYB; signaling pathways, including the Notch pathway; as well as inputs from adjacent tissues, coordinates the entire process [38]. In brief, RUNX1, a key regulator of hematopoiesis, promotes the expression of genes required for endothelial cells to acquire hematopoietic identity [39], whereas c-MYB supports the survival and proliferation of emerging HSCs [40]. Globin transcription factor-2 (GATA-2) further facilitates the process by promoting EHT, even though it is not directly required for the specification of the hemogenic endothelium [41]. Accumulating evidence so far suggests that these transcription factors are regulated by m^6^A modification, which acts as an additional layer of epitranscriptomic control over the expression of key EHT regulators and plays a pivotal role in the successful generation of HSCs.

Specifically, Zhang and colleagues [42] showed that reduced METTL3 protein, and hence reduced m^6^A levels in zebrafish embryos, lead to EHT disruption and eventually to complete loss of specialized blood cells. Interestingly, epitranscriptome mapping revealed that the lack of HSC emergence was caused by reduced m^6^A mRNA methylation of neurogenic locus notch homolog protein 1 (Notch1) and of other arterial endothelial genes, which are normally under YTHDF2-mediated degradation [42]. Consistently, lower levels of the HSPC markers RUNX1 and c-myb were detected, and analogous results were obtained by *ythdf2* zebrafish morphants [42]. Additionally, m^6^A implication in EHT regulation was confirmed in Mettl3-knockdown hematopoietic stem and progenitor cells acquired from the aorta-gonad-mesonephros region (AGM) of mice [42]. Employment of m^6^A-RNA immunoprecipitation (RIP)-qPCR assays and conventional qPCR analysis revealed that Notch1 is m^6^A-targeted, and its expression is enhanced following Mettl3 knockdown. Furthermore, in vivo experiments using the Cre/LoxP system to knockout the expression of Mettl3 specifically in endothelial cells of the mouse AGM region provided consistent results [42]. Collectively, METTL3 and YTHDF2 operate synergistically to suppress the Notch signaling pathway and thus affect the EHT process [42]. Similarly, a study by Lv et al. [43] showed that METTL3 depletion in vascular endothelial cells of mouse AGM leads to a significant suppression of HSPC function. Moreover, there seems to be an interplay between epigenetic and epitranscriptomic mechanisms that are involved in EHT, such as microRNAs (miRNAs), known to affect HSC production and control m^6^A levels via modulating METTL3 binding to mRNAs [44]. The above findings clearly show that m^6^A modification plays an essential role during vertebrate definitive hematopoiesis by fine-tuning the expression of key transcription factors such as RUNX1, c-MYB, and GATA2 as well as signaling molecules like Notch1 to ensure precise HSC emergence [44].

### 2.2. HSC Self-Renewal and Maintenance

Hematopoietic stem cells are multipotent precursors that have the dual ability for self-renewal and for giving rise to myeloid, lymphoid, and megakaryocytic-erythroid cell lineages throughout life [32]. This equilibrium is maintained by cell fate decisions made during cellular division [32]. It has not been fully elucidated yet which signals determine whether a cellular division will result in lineage commitment and differentiation or in self-renewal. The expansion and maintenance of HSC self-renewal are coordinated by signaling pathways, including Notch [45], Wnt [46], BMP [47], and Hedgehog [48] pathways. For instance, Notch signaling promotes HSC quiescence and prevents premature HSC differentiation, whereas canonical Wnt signaling is activated by extracellular proteins and supports symmetric self-renewal divisions. These signaling pathways are regulated by both intrinsic and extrinsic mechanisms [49]. Intrinsic mechanisms include transcription factors, such as RUNX1, GATA-2, c-MYB, and T-cell acute lymphocytic leukemia protein 1 (TAL1), as well as epigenetic and epitranscriptomic regulators, including DNA methylation enzymes and m^6^A-related proteins. Extrinsic mechanisms are mediated by the HSC niche, which provides physical interactions, growth factors, and chemical cues that trigger the different signaling pathways regulating HSC fate decisions [49]. According to recent studies, m^6^A modification and its regulators seem to be key participants in HSC homeostasis [50,51,52,53].

Among the m^6^A readers, YTHDF2, which mediates targeted m^6^A-mRNA degradation, was reported to uniquely influence HSC regeneration and expansion. Recent studies showed that the loss of YTHDF2 leads to increased proliferation of HSC without altering lineage bias [50]. Particularly, Wang et al. [50] exploited Cre/LoxP technology to create hematopoietic-specific *Ythdf2*-knockout mice. According to their study, *Ythdf2* expression in HSCs expedites the decay of m^6^A-marked mRNAs of Wnt target genes, leading to suppression of Wnt signaling at steady state. Given the fact that aberrant activation of Wnt signaling leads to an increase in the number of HSCs with lower functional response to hematological stressors, YTHDF2 probably has a protective role. Interestingly, upon YTHDF2 loss, not only was the number of HSCs increased, but so was the regenerative capacity of HSCs under stress circumstances [50]. Furthermore, Mapperley et al. [51] showed that YTHDF2 protects HSCs from proinflammatory signals, which cause excessive proliferation and promote myeloid-biased differentiation. These data also suggest that *Ythdf2* deletion results in the expansion of HSCs and multipotent progenitors (MPPs) [51]. Similar observations were made by Li et al. [52] after the depletion of YTHDF2 in mouse HSPCs and human umbilical cord blood HSCs. Data obtained by Ythdf2 knockout mice indicated that YTHDF2 normally suppresses HSC self-renewal through RNA degradation. Furthermore, m^6^A mapping revealed that mRNAs of transcription factors important for stem cell self-renewal, such as TAL1 and GATA-2, are m^6^A-labeled and recognized by YTHDF2, which drives them to decay sites [52]. Moreover, Ythdf2 knockdown in human umbilical cord blood HSCs led to decreased apoptotic rates and enhanced expansion ex vivo, providing possible new strategies for future stem cell-based treatments [52]. Reader control of hematopoiesis extends beyond YTHDF2. Another YTH domain containing an m^6^A reader, the YTHDF3, cooperates with METTL3 to recognize and promote the translation of the m^6^A-modified Cyclin D1 mRNA, which is a key regulator of cell cycle progression, thereby supporting HSC self-renewal [54]. In addition to cytoplasmic readers, the nuclear m^6^A reader YTHDC1 is also essential for normal hematopoiesis and HSPC maintenance according to recent studies, partly by regulating miRNA maturation [53,55]. Loss of YTHDC1 results in HSC apoptosis, highlighting its critical role in HSC survival [55]. In line with the previous studies, Yin et al. [31] proposed that m^6^A could be considered as a quality control system for the preservation of HSC integrity. Their findings suggest that the reader protein IGF2BP2 plays a critical role in maintaining HSC stemness by regulating the expression state of m^6^A-methylated mRNAs as well as mitochondrial activity of HSCs [31]. Taken together, these studies suggest that the m^6^A readers cooperatively regulate transcript stability, signaling, and metabolic homeostasis to preserve HSC self-renewal and functional integrity.

Other m^6^A regulators whose role has been studied in mammalian hematopoietic development include the m^6^A writers METTL3 and METTL14. Their role in the control of HSC self-renewal was assessed in adult mouse bone marrow utilizing Cre/LoxP technology to create *Mettl3*-/*Mettl14*-deficient mice [56]. Deletion of these genes led to the accumulation of HSCs and a reduction of their self-renewal capacity. Specifically, it was shown that METTL3 enhances the expression of genes related to HSC quiescence, maintenance, and self-renewal, such as nuclear receptor subfamily 4 group A member 2 (Nr4a2), cyclin-dependent kinase inhibitor 1A (CDK1A), Bmi-1 proto-oncogene polycomb ring finger, and PR domain-containing 16 (Prdm16) [56]. Notably, qPCR analysis showed no difference in the expression of genes related to HSC differentiation, such as Ikaros, PU.1, and Mef2c [56]. Consistent with these results, deletion of *Mettl3* in adult mouse HSCs, and therefore lack of m^6^A, leads to an accumulation of HSCs in the bone marrow while having no important effect on HSC self-renewal and quiescence [29]. On the contrary, a recent study demonstrated that the previously identified accumulated HSCs are actually blocked HSC-multipotent progenitor-like cells, which fail to differentiate due to loss of *Myc*, which controls HSC symmetric commitment and is normally under m^6^A regulation [3]. Moreover, it was shown that *Mettl3* knock-out HSCs are less quiescent and have increased metabolism, as well as mitochondrial activity, indicating that m^6^A is essential for HSC quiescence and self-renewal [3]. In addition, loss of METTL3-mediated m^6^A modification decreases HSC quiescence, increases metabolism and mitochondrial activity, and enhances the formation of endogenous double-stranded RNAs and abnormal stimulation of melanoma differentiation-associated protein 5 (MDA5)-retinoic acid-inducible gene I (RIG-I), protein kinase R (PKR)-eukaryotic initiation factor 2α (eIF2α), and 2′,5′-oligoadenylate synthetase/ribonuclease L (OAS-RNase L) pathways in HSPCs, leading to accumulation of dysfunctional immature cells in vitro and in vivo [57]. Finally, m^6^A erasers are also implicated in the regulation of HSC self-renewal. ALKBH5 was found to stabilize transcripts of key metabolic enzymes, such as oxoglutarate dehydrogenase (OGDH) [58]. Loss of ALKBH5 results in destabilization of *Ogdh* mRNA; impaired mitochondrial oxidative phosphorylation and ATP production; and consequently reduced HSC metabolic activity, self-renewal, and long-term repopulation potential [58]. More recently, FTO-mediated regulation of m^6^A levels was shown to be essential for HSC expansion and homing [59].

Taken together, m^6^A RNA modifications and their regulators play a central role in maintaining HSC homeostasis, self-renewal, and functional integrity, as summarized in Table 1. The MTase complex is critical for controlling HSC differentiation and preventing the accumulation of dysfunctional progenitors, while the m^6^A eraser ALKBH5 ensures metabolic stability by regulating key enzymatic transcripts. Finally, m^6^A readers, such as YTHDF2, YTHDF3, YTHDC1, and IGF2BP2, cooperatively regulate transcript stability, signaling pathways, and metabolic activity to preserve HSC quiescence and regenerative capacity. Collectively, these findings highlight that m^6^A-mediated epitranscriptomic regulation functions as a quality control system, coordinating intrinsic and extrinsic signals to safeguard HSC fate decisions, lineage commitment, and stress response.

### 2.3. Erythropoiesis

Hematopoietic stem cells replenish all blood cell types on a continuous basis through a series of differentiation stages and recurrent cellular divisions that entail the production of lineage-committed progenitors. Daily, the erythroid progenitors expand enormously to generate 200 billion red blood cells in the bone marrow during a stepwise maturation process known as erythropoiesis [60]. Erythropoiesis is controlled by a network of factors, such as oxygen sensors, cytokines, and regulators of iron metabolism [60]. Any abnormalities in critical components of this process can result in severe disorders such as anemia or myelodysplastic syndromes [60]. Briefly, HSCs initially give rise to multipotent progenitors (MPPs), which in turn give rise either to common myeloid precursors (CMPs) or common lymphoid precursors (CLPs), through cytokine signaling and activation of several transcription factors [61,62]. MPPs differentiate into CLPs that originate lymphocytes and natural killer cells that rely mainly on activation by SPI1, Ikaros, and GATA-3 transcription factors [61,63,64,65]. MPP fate-decision differentiation into CMP generates granulocyte-macrophage (GMP) and megakaryocyte-erythroid progenitors (MEPs), modulated by SPI1 and GATA-1 [61,65]. The differentiation of CMP relies on the secretion of granulocyte-macrophage-colony stimulating factor (GM-CSF) and subsequently on macrophage-colony stimulating factor (M-CSF), which regulates the differentiation of monocytes/macrophages, while granulocyte-colony stimulating factor (G-CSF) regulates the differentiation of neutrophils, basophils, and eosinophils. During erythropoiesis, MEP differentiates into burst-forming unit-erythroid (BFU-E) and, finally, into colony-forming unit-erythroid (CFU-E) [60]. This process is modulated by soluble factors such as erythropoietin (EPO), stem cell factor (SCF), interleukin-3 (IL-3), and interleukin-6 (IL-6), as well as by the activation of GATA-1, signal transducer and activator of transcription 5 (STAT-5), and Krüppel-like factor-1 (KLF-1) pathways [60].

Despite the extensive knowledge in this field, many of the regulatory features that control HSC differentiation, as well as the production and maturation of erythrocytes, remain unknown. Numerous studies have shown that m^6^A RNA methylation plays a pivotal role in normal and pathological erythropoiesis [3,66,67,68]. Epitranscriptomic sequencing, either with m^6^A-sequencing or by MeRIP-seq technology, has enabled the mapping of m^6^A sites on the mRNA molecules of mammalian cells. Notably, transcripts of transcription factors that coordinate erythroid differentiation, such as KLF1, GATA-1, GATA-2, Friend leukemia integration 1 transcription factor (FLI1), and myeloproliferative leukemia protein (MPL), and of genes involved in erythroid disorders, were found to be enriched in m^6^A RNA methylation [66]. KLF1 and GATA-1 are known to work together, as well as with other cofactors and chromatin modifiers, to drive the erythroid transcriptional program [66]. Low levels or complete loss of GATA-1 have been linked with impaired terminal erythroid differentiation and anemia development [69]. KLF1 is a principal regulator of erythropoiesis that directly regulates the expression of genes involved in α and β-globin and iron-bound heme biosynthesis [70]. Particularly, KLF1 has been demonstrated to activate the expression of β-globin and regulate the expression of genes that participate in the production of hemoglobin molecules, such as B-cell lymphoma/leukemia 11A (BCL11A), which is known to control the production of fetal γ-globin [69]. Erythropoiesis is substantially hampered upon loss of KLF1 [70]. As a result, the reduction or absence of β-globin chain production leads to the accumulation of an excess of α-globin chains, which precipitate, forming insoluble aggregates in the red blood cell precursors, leading to apoptosis at the polychromatophilic erythroblast stage [71].

Genome-wide CRISPR screening in human erythroleukemia (HEL) cells first established the requirement of the MTase complex in erythropoiesis [66]. Specifically, transduction of HEL cells with lentiviral sgRNA reagents, followed by sorting of CD235^-^ cells out of the population of the outgrown HEL cells, disclosed that METTL3, METTL14, and WTAP were among the genes required for normal erythroid differentiation. Next, CRISPR-Cas9 gene targeting was carried out to knock out each of the main components of the MTase complex and thus to elicit a reduction in global m^6^A levels. The data documented that elimination of the m^6^A MTase complex results in erythroid differentiation blockage without affecting the development of other non-lymphoid cell lineages. Further analysis showed that m^6^A marking enhanced the translation of genes involved in erythroblast differentiation and heme biosynthesis as well as of genes that constitute the H3K4 MTase network [66]. Intriguingly, METTL3 and WTAP knockdown led to the loss of H3K4me3 marking in KLF1-bound promoters, leading to decreased binding capacity [66]. Similar results were obtained following m^6^A loss in human adult bone marrow HSPCs. Knockdown of MTase complex components revealed that their activity is required for early erythropoiesis but not for megakaryocytic differentiation [66]. Also, it was revealed that WTAP enhances the translation of polyadenylate-binding protein 1 and 4 (PABPC1, PABPC4), which promote the expression of β-globin in human HSPCs. Taken together, the data suggest that m^6^A modification is implicated in the regulation of erythropoiesis by maintaining the H3K4me3 marking at erythroid gene targets and promoting erythroid lineage development [66].

Beyond these m^6^A writers, METTL16, which preferentially deposits m^6^A marks in structured RNAs, has emerged as an additional m^6^A methyltransferase with distinct targets relevant to erythroid homeostasis [68,72]. Notably, METTL16 targets the methionine adenosytransferase 2A (MAT2A) transcript, which is responsible for producing S-adenosylmethionine (SAM) [72]. SAM is a universal methyl donor essential for various methylation reactions in cells. By modulating MAT2A expression, METTL16 indirectly influences SAM levels, thereby impacting global methylation capacity and cellular methylation-dependent processes [72]. Because SAM is required for METTL3-mediated m^6^A installation and for histone methyltransferases that deposit marks such as H3K4me3, METTL16 can indirectly influence both RNA methylation and chromatin states that are critical for erythroid gene expression. Yoshinaga et al. [68] showed that METTL16 depletion in erythroblasts causes loss of m^6^A on DNA-repair transcripts, accumulation of DNA damage, and increased apoptosis [68,73]. Interestingly, supplementing cells with SAM can mitigate some defects, demonstrating that METTL16’s control of SAM contributes functionally to erythropoiesis [68]. Collectively, these findings position METTL16 as a crucial regulator of erythropoiesis.

The significance of the m^6^A writers in erythropoiesis is further highlighted in the regulation of MYC, a proto-oncogenic protein known to control the balance between HSC self-renewal and differentiation. During terminal erythroid differentiation, its protein levels are rapidly decreased [74]. Transcripts of c-MYC, BCL-2, and phosphatase and tensin homolog (PTEN) are enriched in m^6^A marks [74]. Specifically, METTL3-mediated m^6^A targeting of these transcripts promotes their translation and subsequently blocks differentiation of HSPCs [74]. Importantly, the functional outcome of these modifications depends on their positional placement within the sequence of mRNAs. In the case of c-MYC, m^6^A methylation within a 250 bp region at the 3′ end of the coding sequence and 3′ UTR enhances transcript stability and translation through the recognition by the reader family IGF2BPs and YTHDF1, thereby sustaining HSC self-renewal [75,76,77]. Similarly, m^6^A in the 3′ UTRs of BCL-2 and PTEN promotes efficient translation and stability, further supporting survival and blocking differentiation of HSPCs [54,77]. Although a complete positional mapping of these transcripts remains unresolved, accumulating evidence underscores that the location of m^6^A marks is critical in dictating their fate during hematopoietic regulation.

Furthermore, key components of the MTase complex seem to regulate *Myc* RNA stability and expression in HSPCs [3]. Specifically, siRNA depletion of *Mettl3* in normal murine HSPCs resulted in decreased MYC protein levels and blockage of HSC symmetric commitment, while in *Mettl3*-knockout mice, accumulation of immature erythroblasts, leading to spleen enlargement and disruption of its architecture, was observed. METTL14 was found to be negatively regulated by the SPI1 transcription factor both in normal and pathological conditions such as acute myeloid leukemia [78]. For the first time, Weng and coworkers [78] described the signaling axis SPI1-METTL14-MYB/MYC in normal myelopoiesis and leukemogenesis and proposed that METTL14 could be a new therapeutic target to treat acute myeloid leukemia (AML). This view is further supported by the fact that upon its deletion, HSCs are expanded, while AML malignant stem cells are selectively compromised [79].

Moreover, c-Myc expression is regulated by the RBM15, which was recently identified as a component of the MTase complex [80]. RBM15 was found to be highly expressed in the hematopoietic system [80,81]. Particularly, in vitro studies of cultured AML cells showed that knockdown of RBM15 leads to impaired differentiation and increased apoptosis rate. Also, knockout mouse studies revealed that RBM15 is implicated in HSC expansion and differentiation, probably by affecting Notch signaling and c-Myc expression, respectively [81]. Furthermore, it was found that the expression of METTL14 and FTO is significantly lower in mature hematopoietic cells. No significant expression pattern was found for METTL3, WTAP, or ALKBH5. Consistent with these data, recent studies showed that ALKBH5 is not required for maintaining the function of normal HSCs [82,83]. Moreover, METTL14 knockdown in normal CD34^+^ cells resulted in enhanced myeloid differentiation, colony formation inhibition, and decreased expression of the oncogenic transcription factors MYB and MYC [3]. Normally, METTL14 and FTO were found to promote the stability of MYC mRNA via IGF2BP-mediated regulation of its expression [3]. Both YTHDF2 and IGF2BPs act as regulators of mRNA stability. However, contrary to the mRNA degradation-promoting function of YTHDF2, IGF2BPs were reported to promote mRNA stability [77]. In vivo models of murine AML and analysis of primary samples from patients with AML revealed that the RNA-binding protein Y-box binding protein 1 (YBX1) affects the stability of m^6^A-enriched transcripts of apoptotic genes, such as *Bcl2* and *c-Myc*, by interacting with IGF2BPs [84]. However, two independent studies demonstrated that YBX1 does not affect normal hematopoiesis or the function of HSCs [73,84]. It is also noteworthy that a recent study in cancer cells documented that the m^6^A reader protein YTHDF1 affects transferrin receptor-mediated iron metabolism [85].

Collectively, these studies highlight that m^6^A RNA methylation is a critical regulator of erythropoiesis, controlling HSPC differentiation, erythroid lineage commitment, and terminal maturation. m^6^A writers, such as METTL3, METTL14, WTAP, and METTL16, as well as m^6^A readers (YTHDF1, IGF2BPs) coordinate the differentiation of HSPCs into erythroid lineages by modulating factors like GATA-1, KLF1, and MYC as well as ensuring proper heme biosynthesis and globin production. Dysregulation of this epitranscriptomic network can lead to impaired erythroid maturation, anemia, or hematologic malignancies, emphasizing the therapeutic potential of targeting m^6^A pathways in blood disorders.

### 2.4. The Role of m^6^A Modification in Malignant Myeloid Hematopoiesis

**Figure 2:**
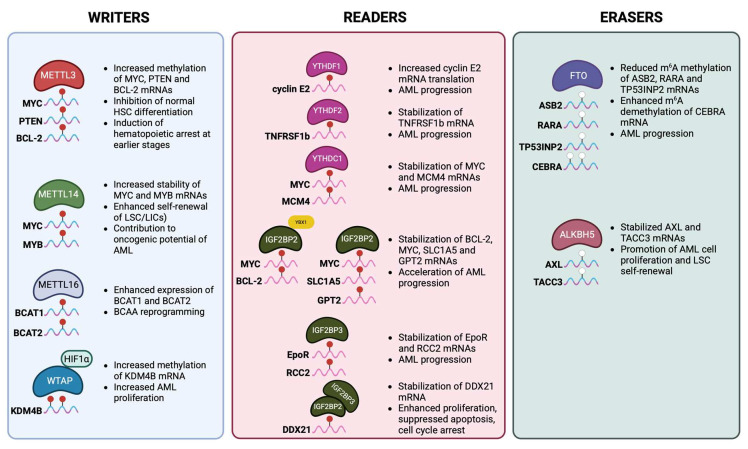
The basic m^6^A modifications occurring in acute myeloid leukemia (AML). Methyltransferases, demethylases, and reader proteins contribute to AML progression by regulating the stability and/or decay of different crucial mRNA transcripts, thus interfering with translation and eventual protein production. MYC, myelocytomatosis oncogene (also known as c-Myc); PTEN, phosphatase and tensin homolog; BCL-2, B-cell lymphoma 2; MYB, myeloblastosis oncogene; LSC, leukemia stem cell; LIC, leukemia-initiating cell; BCAA, branched-chain amino acid; BCAT1/2, branched-chain amino acid transaminase 1/2; HIF1α, hypoxia-inducible factor 1-alpha; KDM4B, lysine demethylase 4B; TNFRSF1b, tumor necrosis factor receptor superfamily member 1b; MCM4, minichromosome maintenance complex component 4; YBX1, Y box-binding protein 1; SLC1A5, solute carrier family 1 member 5; GPT2, glutamic–pyruvic transaminase 2; EpoR, erythropoietin receptor; RCC2, regulator of chromosome condensation 2; DDX21, DEAD-box helicase 21; ASB2, ankyrin repeat and SOCS box containing 2; RARA, retinoic acid receptor alpha; TP53INP2, tumor protein P53 inducible nuclear protein 2; CEBPA, CCAAT enhancer-binding protein alpha; AXL, AXL receptor tyrosine kinase; TACC3, transforming acidic coiled-coil-containing protein 3. Adapted from Chen et al. [93]. Created in Biorender. Vasilopoulou, A-G. (2025) https://BioRender.com/nk1sx3l (accessed on 26 August 2025).

Studies investigating the role of m^6^A modification in hematologic malignancies have demonstrated that it profoundly affects gene expression, signaling networks, and cell fate decisions. Acute myeloid leukemia, the most prevalent and aggressive form of acute leukemia in adults, is characterized by the abnormal clonal proliferation of primitive HSPCs. Dysregulation of m^6^A marking was found to significantly alter gene expression programs, thereby sustaining leukemia stem cell (LSC) self-renewal, blocking normal HSC differentiation, and driving leukemogenesis. The basic m^6^A modifications that have been reported in AML are summarized in Figure 2.

Aberrant expression of m^6^A writers in AML compared to normal HSCs alters the methylation levels of key transcripts, promoting malignant transformation. In AML patients, METTL3 was found to be upregulated at diagnosis and during relapse compared to patients in complete remission [86]. This overexpression of METTL3 results in increased methylation of MYC, PTEN, and BCL-2 transcripts, and, thus, upregulation of their expression and inhibition of normal HSC differentiation [74]. Clinical and translation studies further link upregulated METTL3 expression to the development of AML chemoresistance by regulating the homing and engraftment of AML cells [86]. Similarly, METTL14 was found to contribute to AML development by stabilizing transcripts of MYC and MYB, which leads to enhanced self-renewal of LSCs. Furthermore, Han et al. [87] showed that METTL16 is essential for survival and stemness of the AML cells by regulating the branched-chain amino acid (BCAA) metabolic pathway and particularly the expression of branched-chain amino acid transaminases 1 and 2 (BCAT1 and BCAT2) [87]. BCAA metabolism sustains the anabolic demands of LSCs, thereby supporting their rapid proliferation [87]. In AML, WTAP is also activated by the hypoxia-inducible factor 1α (HIF1α) and consequently increases the m^6^A methylation of lysine-specific demethylase 4B (KDM4B) transcripts [88]. This modification stabilizes KDM4B transcripts and further enhances the proliferation of LSCs [88].

m^6^A demethylases were also found to be upregulated in AML, contributing to its pathogenesis. This upregulation leads to decreased m^6^A methylation levels of mRNAs that drive myeloid differentiation, including retinoic acid receptor alpha (RARA) and ankyrin repeat and SOCS box protein 2 (ASB2) [89]. More specifically, increased expression of FTO results in destabilization and reduced translation of these transcripts, thereby blocking normal differentiation and promoting AML progression [89]. Additionally, ALKBH5 is required for LSC maintenance and function during the leukemogenesis of AML [82]. By erasing m^6^A marks on specific target mRNAs, ALKBH5 protects oncogenic transcripts from YTHDF-mediated decay, thereby supporting AML pathogenesis [82].

m^6^A reader proteins also play an important role in AML by modulating the fate of the modified mRNAs and thereby influencing leukemogenesis. The cytoplasmic readers YTHDF1 and YTHDF2 promote AML progression by enhancing the translation of cyclin E2 and inhibiting the expression of TNF-α, respectively [79]. Increased expression of cyclin E2 promotes LSC proliferation, whereas downregulation of TNF-α results in suppressed apoptosis, further supporting LSC self-renewal. Nuclear m^6^A readers, including YTHDC1 and HNRNPC, regulate pre-mRNA splicing and processing of genes involved in cell cycle control and differentiation, such as MYC, thereby supporting AML cell survival [53,90]. Additionally, the IGF2BP family (IGF2BP1–3) stabilizes oncogenic mRNAs such as MYC and BCL-2, enhancing their translation and sustaining LSC self-renewal [76,91]. Taken together, these readers ensure that oncogenic transcripts are efficiently translated and protected from decay, reinforcing the block in differentiation and promoting leukemogenesis.

**Table 1:** The role of different m^6^A regulators in the renewal, differentiation, and maintenance of HSCs.

m^6^A Regulator	Type	Role in HSCs	Effect on Self-Renewal, Differentiation and Maintainance	References
METTL3	Writer (m^6^A methyltransferase)	Catalyzes m^6^A deposition with METTL14	Essential for HSC self-renewal; loss leads to impaired HSC maintenance, defective differentiation, and bone marrow failure	[56,57]
METTL14	Writer (METTL3 partner)	Stabilizes METTL3 and regulates target mRNAs	Required for HSC self-renewal; deletion causes loss of HSC quiescence and differentiation bias	[29,56]
WTAP	Writer complex component	Regulatory subunit for METTL3/METTL14	Contributes to HSC survival and lineage commitment	[66]
FTO	Eraser (demethylase)	Removes m^6^A from target transcripts	Overexpression reduces differentiation, maintaining stemness; depletion promotes myeloid differentiation	[59]
ALKBH5	Eraser (demethylase)	Regulates mRNA stability and splicing	Helps maintain stem cell pool under stress; linked to leukemogenesis	[58]
YTDHF1	Reader (cytoplasmic)	Enhances translation of m^6^A-marked transcripts	Promotes lineage-specific differentiation by boosting translation of key regulators	[54]
YTDHF2	Reader (cytoplasmic)	Mediates degradation of m^6^A-modified RNAs	Critical for HSC self-renewal; deletion leads to increased HSC numbers but reduced long-term function	[50,51,52]
YTHDF3	Reader (cooperates with YTHDF1/2)	Balances translation vs decay	Coordinates with YTHDF1/2 in HSC maintenance	[54]
YTHDC1	Reader (nuclear)	Controls mRNA splicing and export	Ensures proper transcript processing in HSC renewal; deletion causes impaired hematopoiesis	[53,55]
IGF2BP1/2/3	Readers (stabilizers)	Bind and stabilize m^6^A-marked mRNAs	Enhance expression of stemness-associated genes, supporting HSC maintenance and survival	[31]
HNRNPC	Reader (nuclear)	Binds to m^6^A-switch structures	Controls transcript fate, influencing HSC homeostasis	[90]

### 2.5. Ineffective Erythropoiesis and Apoptosis

Apoptosis and autophagy are cell death machineries widely associated with ineffective erythropoiesis and blood cancers. More specifically, apoptosis eliminates defective or excess erythroid progenitors, preventing accumulation of abnormal cells, while autophagy degrades and recycles damaged organelles and proteins, facilitating erythroblast maturation and enucleation. Dysregulation of either process can lead to ineffective erythropoiesis, as observed in disorders such as β-thalassemia and myelodysplastic syndromes, and contributes to hematologic malignancies [92,93]. Notably, transcripts of pro-apoptotic and apoptotic genes, for instance *MYC*, *BCL-2*, and *PTEN*, as well as autophagy-related genes, for instance, the autophagy-related 7 gene, *ATG7*, were found to be enriched in m^6^A marks [25,94,95]. The presence or absence of m^6^A on these RNAs substantially alters their translation, stability, or decay with direct consequences for cell survival.

Several studies have shown that METTL3, which is the core component of the MTase complex, plays an important role in apoptosis mainly by regulating the expression of Bcl-2 [96]. Specifically, METTL3 promotes the translation of pro-survival mRNAs in myeloid cells, including *MYC* and *BCL-2*, thereby favoring cell survival and blocking differentiation. Additionally, the demethylases FTO and ALKBH5 primarily function to prevent apoptosis. FTO knockdown in AML cell lines led to increased m^6^A levels of MYC mRNA, promoting its degradation and thus enhancing apoptosis [97]. m^6^A regulators are also involved in the autophagy pathway [98,99]. Specifically, it has been demonstrated that m^6^A RNA methylation controls the expression of autophagy-related gene (ATG) transcripts [98,99]. The m^6^A-marked ATG transcripts are under YTHDF2-mediated degradation and, therefore, autophagy is inhibited [98]. Further research is needed to decipher how the relationship between m^6^A and cell death could be implicated in the pathophysiology of hematologic diseases.

### 2.6. The Role of m^6^A Modification in Thalassemia

**Figure 3:**
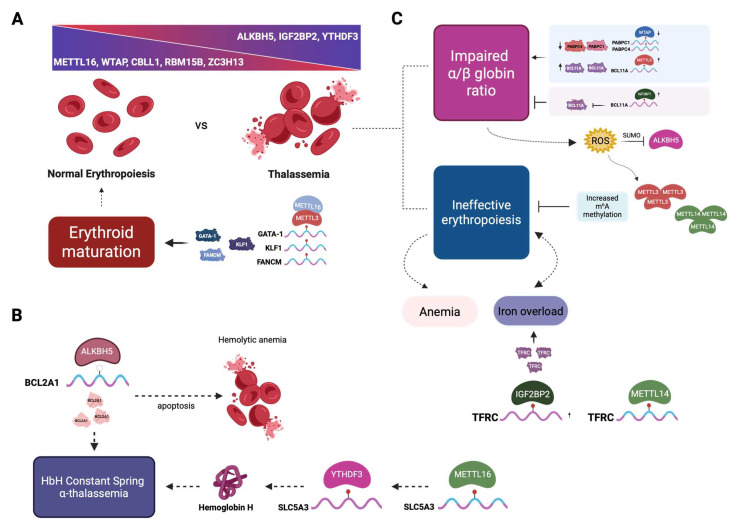
The impact of m^6^A modifications in thalassemia and their role in the disease pathophysiology. (**A**). Normal erythroblasts demonstrate high levels of METTL16, WTAP, CBLL1, RBM15B, and ZC3H13, while erythroblasts of HbH disease Constant Spring α-thalassemia overexpress ALKBH5, IGF2BP2, and YTHDF3. Furthermore, in normal erythropoiesis, increased expression of METTL16 and METTL3 positively regulates the production of the erythroid regulatory proteins GATA-1, KLF1, and FANCM, preventing DNA damage and apoptosis, thus ensuring efficient erythroid maturation. (**B**). Ιn HbH Constant Spring α-thalassemia, the anti-apoptotic protein BCL2A1 is downregulated via the action of ALKBH5, and as a result, apoptosis remains uninhibited, leading eventually to hemolytic anemia. Furthermore, it has been speculated that the expression of Hemoglobin H and hence the disease phenotype is affected by the modification of SLC5A3 mRNA by METTL16 and its sequential reading by YTHDF3. (**C**). On the other hand, the β-thalassemia phenotype is primarily the result of impaired α/β ratio of globin synthesis and ineffective erythropoiesis. In the former case, reduced m^6^A methylation of PABPC1 and PABPC4 mRNAs by WTAP leads to reduced PABPC1 and PABPC4 protein levels and deteriorates the detrimental effects of the impaired α/β ratio since these two proteins affect mRNA isoform diversity during erythropoiesis and stabilize a subset of erythroid mRNAs, shielding them from degradation, a crucial feature for terminal erythroid maturation [101]. Furthermore, elevated METTL3 expression stabilizes BCL11A, a major γ-globin repressor, inhibiting the capacity for the beneficial reactivation of the fetal γ-globin genes for HbF production. Interestingly, overexpression of the reader IGF2BP1 has been recently shown to induce fetal hemoglobin in cultured erythroid cells from patients with thalassemia or sickle cell disease, reducing the α/β globin imbalance, probably by inhibiting the translation of BCL11A mRNA via binding [102]. With regards to ineffective erythropoiesis, as a result of the impaired α/β globin ratio, there is an increased precipitation of excess α-globins that leads to the destruction of erythroid precursors, releasing free iron that ultimately leads to ROS production. In turn, ROS enhances the expression of METTL3 and METTL14, also inhibiting the demethylase activity of ALKBH5 via SUMO. Elevated levels of the aforementioned methyltransferases result in increased m^6^A methylation levels that demonstrate a protective role for cell genome integrity, reducing ineffective erythropoiesis and thus ameliorating the clinical manifestations of thalassemia. Lastly, elevated METTL14 expression in thalassemia leads to increased IGF2BP2-binding TFRC mRNAs, contributing to iron overload. KLF1; Krüppel-like factor 1; FANCM, FA complementation group M; PABPC, poly(A)-binding protein; TFRC, transferrin receptor; SUMO, small ubiquitin-like modifier; ROS, reactive oxygen species; BCL-2, B-cell lymphoma 2. Partially adapted from Zheng et al. [103]. Created in BioRender. Vasilopoulou, A-G. (2025) https://BioRender.com/2uz1er2 (accessed on 26 August 2025).

Recent studies revealed that m^6^A RNA modification also affects the expression of genes involved in hemoglobin production, heme biosynthesis, iron metabolism, and cell death mechanisms, such as apoptosis and autophagy [85,97]. These findings imply that m^6^A modification could also be a key participant in the pathogenesis of thalassemic hematopoiesis (Figure 3). Recently, Ruan et al. [100] demonstrated for the first time that m^6^A modification is linked to the phenotype of the Hemoglobin H Constant Spring variant, a severe form of α-thalassemia caused by mutations of the duplicated α-globin genes (--/α^CS^α) that affect the production of α-globin chains. For their study, immature erythrocytes were derived from the peripheral blood of 16 thalassemia patients and 15 healthy individuals. Data acquired from qRT-PCR and m^6^A mRNA and lncRNA epitranscriptomic microarrays revealed that the expression pattern of m^6^A-related factors is significantly different between the two groups. Specifically, they discovered that the expression of the MTase complex components METTL16, WTAP, CBLL1, RBM15B, and ZC3H13 was lower in this type of α-thalassemia compared to normal erythroid cells, whereas the eraser protein ALKBH5, as well as the readers IGF2BP2 and YTHDF3, were overexpressed (Figure 3A). However, these findings were based on transcriptomic analyses and the decreased expression of the above genes reflects changes at the mRNA level. The impact of these transcript-level alterations on protein abundance remains unverified, as proteomic assessment was beyond the scope of that study and no published data are currently available addressing this issue. Furthermore, the authors employed m^6^A mapping to identify the differentially hypo-m^6^A-methylated transcripts in the two cohorts. Interestingly, the transcript of the anti-apoptotic protein BCL2A1 was downregulated in thalassemia patients. Therefore, in HbH Constant Spring α-thalassemia, apoptosis remains uninhibited as a result of the decreased expression of the BCL2A1, eventually leading eventually to hemolytic anemia (Figure 3B). According to their findings, the ALKBH5-mediated alteration of the m^6^A methylation status of BCL2A1 contributes to the pathogenesis of α-thalassemia. However, the exact mechanism by which m^6^A modification regulates BCL2A1 expression remains to be elucidated.

Further evidence for the role of METTL16 in the pathogenesis of Hemoglobin H disease was provided recently [16], where the METTL16, YTHDF3, and solute carrier family 5 member 3 (SLC5A3) mRNAs were downregulated in HbH patients. Modification of the METTL16 levels in human erythroleukemia K562 cells provided evidence that METTL16 affects the expression of hemoglobin via the modification of SLC5A3 mRNA and its sequential reading by YTHDF3 [16], as shown in Figure 3B. Further research is needed to elucidate the mechanisms by which m^6^A modification influences thalassemic hematopoiesis and to explore potential therapeutic strategies targeting these pathways.

## 3. Future Perspectives

Over the past decade, a significant number of studies have confirmed the importance of m^6^A RNA modification in normal hematopoiesis and the pathogenesis of hematological malignancies, particularly AML. However, there is limited knowledge regarding the role of m^6^A modification in non-malignant erythroid disorders, such as the thalassemia syndromes. Thalassemia, an autosomal recessive disease, is characterized by reduced or absent synthesis of the globin chains and is strongly influenced by genetic modifiers and epigenetic regulation. To date, there are only two studies [16,100] that have demonstrated the significant role of m^6^A modification in α-thalassemia, suggesting that altered methylation may influence globin mRNA stability and ineffective erythropoiesis. Moreover, m^6^A regulators modulate the expression of genes involved in heme biosynthesis, iron metabolism, and cell death mechanisms. Therefore, elucidating whether m^6^A modification contributes to other forms of thalassemia is critical, as such insights could be leveraged to reduce ineffective erythropoiesis and enhance globin mRNA stability.

Furthermore, several fundamental questions regarding the molecular basis of m^6^A are yet to be addressed, including m^6^A stoichiometry and topology as well as regulation of the MTase complex [104]. Further research is needed to elucidate the exact function of each component of the m^6^A regulatory network in governing cell fate decisions and to explore the underlying mechanisms by which altered m^6^A levels affect gene expression. Indeed, recent studies have documented that m^6^A modification is a potent inducer of ribosome stalling, leading to ribosome collisions at the m^6^A sites, which in turn is followed by recruitment of the YTHDF reader proteins, promoting RNA degradation [105]. The distribution of m^6^A marks is very consistent among tissues, probably due to the conserved gene architecture [104]. For that reason, any detected differentially regulated m^6^A sites could be used to explore the role of other molecular pathways to m^6^A formation [104,106].

Looking to the future, targeting the m^6^A pathway could be exploited as a new therapeutic tool for treating hematopoietic disorders. However, focus should be given to developing therapeutic strategies that do not have any adverse effects on healthy cells. Lastly, among the remaining major challenges to be addressed is to determine experimentally whether these key m^6^A regulators, the newly discovered coding sequence-m^6^A decay (CMD) pathway [106] and/or the enhancer RNAs (eRNAs) m^6^A modifications [107] could be efficiently targeted by small molecule inhibitors [101] before moving the translation of such findings to the clinical stage.

## 4. Conclusions

This review highlights the role of m^6^A RNA modification in normal hematopoiesis, erythroid differentiation, malignant myeloid hematopoiesis, and its potential implication in thalassemia syndromes. Epitranscriptomic regulation through m^6^A marking has emerged as a fundamental mechanism modulating gene expression in both normal and pathological contexts. In vertebrate hematopoiesis, m^6^A plays an important role in regulating HSC self-renewal, lineage commitment, and terminal erythroid differentiation, in part through affecting the expression and stability of key transcripts such as *MYC, MYB, KLF1*, and GATA1. In malignant myeloid hematopoiesis, particularly in AML, dysregulation of m^6^A regulators promotes leukemic stem cell self-renewal and blocks normal HSC differentiation. While the importance of m^6^A in malignant hematopoiesis is well established, knowledge of the role of m^6^A in non-malignant erythroid disorders, such as thalassemia, remains limited. Early studies on a severe form of α-thalassemia indicated that altered methylation patterns affect globin mRNA stability, ineffective erythropoiesis, and iron metabolism. These findings suggest that m^6^A may play a broader role in modifying disease severity across thalassemia genotypes and related anemias, though systematic investigations are lacking. Taken together, m^6^A RNA modification represents a central epitranscriptomic regulator of hematopoiesis, and its dysregulation not only drives malignant transformation but also holds promise as a potential therapeutic target in non-malignant erythroid disorders, such as thalassemia.

## Data Availability

No new data were created or analyzed in this study. Data sharing is not applicable to this article.
